# Tailoring the Static and Dynamic Mechanical Properties of Tri-Block Copolymers through Molecular Dynamics Simulation

**DOI:** 10.3390/polym8090335

**Published:** 2016-09-19

**Authors:** Zijian Zheng, Hongji Liu, Jianxiang Shen, Jun Liu, Youping Wu, Liqun Zhang

**Affiliations:** 1 Key Laboratory of Beijing City on Preparation and Processing of Novel Polymer Materials, Beijing University of Chemical Technology, 100029 Beijing, China; zhengzijian2008@126.com (Z.Z.); liuhongji1992@gmail.com (H.L.); zhanglq@mail.buct.edu.cn (L.Z.); 2 Beijing Engineering Research Center of Advanced Elastomers, Beijing University of Chemical Technology, 100029 Beijing, China; 3 College of Materials and Textile Engineering, Jiaxing University, 314001 Jiaxing, China; topatom@163.com; 4 Engineering Research Center of Elastomer Materials on Energy Conservation and Resources, Beijing University of Chemical Technology, Ministry of Education, 100029 Beijing, China; 5 State Key Laboratory of Organic-Inorganic Composites, Beijing University of Chemical Technology, 100029 Beijing, China

**Keywords:** self-assembly, tri-block copolymer, molecular dynamics, hysteresis loss

## Abstract

Although the research of the self-assembly of tri-block copolymers has been carried out widely, little attention has been paid to study the mechanical properties and to establish its structure-property relation, which is of utmost significance for its practical applications. Here, we adopt molecular dynamics simulation to study the static and dynamic mechanical properties of the ABA tri-block copolymer, by systematically varying the morphology, the interaction strength between A-A blocks, the temperature, the dynamic shear amplitude and frequency. In our simulation, we set the self-assembled structure formed by A-blocks to be in the glassy state, with the B-blocks in the rubbery state. With the increase of the content of A-blocks, the spherical, cylindrical and lamellar domains are formed, respectively, exhibiting a gradual increase of the stress-strain behavior. During the self-assembly process, the stress-strain curve is as well enhanced. The increase of the interaction strength between A-A blocks improves the stress-strain behavior and reduces the dynamic hysteresis loss. Since the cylindrical domains are randomly dispersed, the stress-strain behavior exhibits the isotropic mechanical property; while for the lamellar domains, the mechanical property seems to be better along the direction perpendicular to than parallel to the lamellar direction. In addition, we observe that with the increase of the dynamic shear amplitude and frequency, the self-assembled domains become broken up, resulting in the decrease of the storage modulus and the increase of the hysteresis loss, which holds the same conclusion for the increase of the temperature. Our work provides some valuable guidance to tune the static and dynamic mechanical properties of ABA tri-block copolymer in the field of various applications.

## 1. Introduction

Block copolymers (such as styrene-butadiene-styrene (SBS) [1] or styrene-isoprene-styrene (SIS) [2]) have gained much interest in the scientific community because of their great potential for a multitude of various applications in the field of advanced materials and biomedicine. The phase diagram of these systems is governed by the polymer chain length, the strength of the phase segregation and the volume fraction of each block [3]. The structure morphologies are varied from spherical clusters [4], to cylindrical columns [5], to gyroidal [6] and lamellar phases [7]. In the case of all three states, the chain ends are confined to the hard glassy minority regions, allowing the rubbery phase to be effectively cross-linked. As both constituents are linked together by the chemical cross-links, this kind of tri-block copolymer can simultaneously combine the mechanical properties of each phase, namely the ductility of the rubbery phase coupled with the toughness of the glassy phase.

Some experimental studies have been carried out to study the self-assemble process of the block copolymer. For instance, the self-assembly of the small molecule surfactant, such as amphiphilic di-block copolymers in aqueous solution, has been extensively studied [8,9,10]. The self-assembled nanostructures, such as spherical micelles, wormlike micelles and vesicles, are governed by the surfactant concentration and its hydrophilic/hydrophobic balance [11]. However, such self-assembly is usually limited to dilute copolymer solutions (<1%), which is a significant disadvantage for potential commercial applications, such as drug delivery and coatings. Besides this, studying the self-assembly structure and its relation with the mechanical properties of block copolymer in the bulk state is also very significant. Adopting small-angle X-ray scattering under deformation, Cohen et al. [12] demonstrate that with increasing strain, the normal to the lamellae tilts away from the stretching direction, whereas the lamellar spacing remains almost constant. Makke et al. [13] further discover the influence of tie and loop molecules on the mechanical properties of lamellar block copolymers, finding that loop chains play the exact same role as tie molecules. Moreover, Qi et al. [14] have observed the structural evolution of the spherical domains during the deformation process, and this evolution is thought to be the primary cause of the dynamic hysteresis loss and the cyclic softening. Later on, Sarva et al. [15] have reported that polyurethane is observed to undergo a transition from a rubbery-regime behavior at the low rates to a glassy-regime behavior at the high rates during the test. However, it is still difficult to obtain a comprehensive picture of how the microstructure of these thermoplastic elastomers (TPEs) determines the response of the macroscopic stress experimentally, and understanding the morphology change at the molecular level during the extension to the large strain is always difficult through experimental analysis.

Computer simulation, as an advanced technique, is playing a more and more important role. Actually, a few simulation studies have been carried out to investigate the self-assembly behavior of phase-separated tri-block copolymers so far [16,17,18]. By adopting the Monte Carlo simulation method, Song et al. [19] have studied the phase behavior of the symmetric ABA tri-block copolymer, which contains a semi-flexible mid-block. The results showed that the increase of the midblock rigidity leads to more chain stretching, which leads to the increase of the individual chain size, configuration transition from loop to bridge and extended loop. Through the Brownian dynamics simulation method, Li et al. [20] have studied the self-assembly behavior of ABA coil–rod–coil tri-block copolymers in a selective solvent. The simulated results revealed that the rod midblock plays an important role in the self-assembly of the copolymers. With the decrease of the segregation strength ε*_RR_* between rod pairs, the aggregate structure first varies from a smectic-like disk shape to a long twisted string micelle, followed by the transition to the aggregates. Besides, the deformation of the tri-block copolymers was also studied by molecular dynamics simulation. For instance, Aoyagi et al. [3] generated a spherical morphology firstly using the self-consistent field (SCF) theory before performing molecular dynamics simulation with the bead-spring model of polymer chains. Uniaxial elongation of short tri-blocks shows failures at the strain of 350% where the minority phase domains are broken up. In order to elucidate the microscopic mechanism, Amanda et al. [21] adopted molecular dynamics simulations to study the plastic deformation of sphere-forming tri-block thermoplastic elastomers. Makke et al. [22] studied the nanoscale buckling in lamellar block copolymers using a coarse-grained molecular dynamics simulation approach. The results revealed that oriented block copolymers exhibit a buckling instability when being submitted to a tensile test perpendicular to the lamellae direction. Moreover, using a novel dissipative particle dynamics (DPD) model, Chantawansri et al. [23,24,25] systematically studied the morphological and mechanical properties of ABA tri-block copolymer. Unfortunately, little research work has been carried out to examine the dynamic hysteresis loss of tri-block copolymer (such as SBS and thermoplastic polyurethane (TPU) [26]) for its potential applications in fuel-saving automobile tires, which is the main motivation of this work.

In this work, we systematically study the static and dynamic mechanical response of a block copolymer model using the coarse-grained molecular dynamics simulation. Here, we focus on the case of the linear symmetric ABA tri-block copolymers, where the hard component is A blocks, while the soft component is B blocks. By choosing various proportions of A blocks, we achieve different morphologies, and the structural evolution during the self-assembly process on the stress-strain behavior is probed. After that, we mainly consider the case of spherical domains. We study the interaction energy between A-A blocks on the static and dynamic properties. Lastly, the temperature and the dynamic shear amplitude on the dynamic mechanical properties are probed. In general, we aim to provide a fundamental understanding about how to manipulate the dynamic mechanical properties of tri-block copolymers.

## 2. Methods

To perform the simulation work, we use the classical coarse-grained molecular dynamics simulation (CGMDS), following the typical bead-spring polymer model developed by Kremer and Grest [27]. We note that although these polymeric chains are rather short, they have already shown the static and dynamic behavior characteristic of long chains. By mapping the coarse-grained model to the real one, each bond corresponds to *n* = 3–6 covalent bonds along the backbone of a realistic chemical chain. Since it is not our aim to study any specific polymer, the mass *m* and diameter σ of each bead is set to be the unit, which indicates that all calculated quantities are dimensionless.

To achieve the glassy domains in the rubbery matrix, we directly reproduce the simulation approach from Aoyagi et al. [3]. The modeled tri-block chain length varies from A_5_B_90_A_5_, A_5_B_20_A_5_ to A_5_B_10_A_5_, corresponding to spherical, cylindrical and lamellar phases, respectively. The number of all beads of each system is set to be 24,000. The non-bonded interaction energy between all polymer beads is modeled through the truncated and shifted Lennard–Jones (LJ) equation as follows:

(1) $$U(r)=\left\{ \begin{array}{ll} 4\varepsilon [{\left(\frac{\sigma }{r}\right)}^{12}-{\left(\frac{\sigma }{r}\right)}^{6}]+C & r<r_{cutoff}\\ \;0 & r\geq r_{cutoff} \end{array}\right.$$

where the LJ interaction is cut off at the distance *r* = *r_cutoff_* and *C* is a constant, which guarantees that the potential energy is continuous at the cutoff distance. *r* is the separation distance between two polymer beads. σ defines the length scale, and ε is the energy scale of our model. The interaction between the adjacent bonded beads is modeled by a stiff finite extensible nonlinear elastic (FENE) potential:

(2) $$U_{FENE}=-0.5kR_{0}^{2}ln[1-{\left(\frac{r}{R_{0}}\right)}^{2}]$$

where *k* = 30 × ε/σ^2^ and *R*_0_ = 1.5 × σ, guaranteeing a certain stiffness of the bonds while avoiding high-frequency modes and chain crossing. We set different interaction strengths and cutoff distances to satisfy the following two points: (1) the domain of the A blocks is formed in the equilibrium state; (2) the block A, corresponding to the hard block, such as the styrene blocks, behaves as a glassy state, while the B blocks, corresponding to the soft bock, such as the butadiene blocks, exhibit a rubbery state. Table 1 shows the list of the parameters used in the interaction potential.

In our simulation, during the equilibration, the canonical ensemble (NVT) is adopted to make sure that the number density of the simulated system is set to be 0.90. We set the simulated temperature equal to T* = 0.4, which is above the glass transition temperature of the B blocks [27] and below that of the A blocks (around 0.46, as shown in Figure S1), by using the Nose-Hoover thermostat and barostat [28]. The velocity-Verlet algorithm is used to integrate the equations of motion with a time step δ*t* = 0.012, where the time is reduced by τ (τ is the unit time of the simulation). The periodic boundary condition is imposed in all three directions. After enough equilibration (1 × 10^8^ molecular dynamics (MD) steps) with the NVT ensemble, we record the change of the potential energy of all three systems in the following 1 × 10^6^ MD steps, as shown in Figure S2a–c. Meanwhile, for all three systems, the change of the mean squared end-to-end distance $R_{\mathrm{end}}^{2}$ and radius of gyration $R_{g}^{2}$ is presented in Figure S2d–f. Obviously, they exhibit small fluctuations, which verify that our simulated systems have been fully and properly equilibrated.

Here, we use the following approach to perform the tensile deformation. All simulated systems are deformed by changing the box length to *L*_0_*a* in the *z* direction and to *L*_0_*a*^−*1/2*^ in the *x* and *y* directions, during which the volume of the simulation box is held constant. The interactions between atoms in the basic cell and image atoms across the cell wall serve to transmit the deformation to the atoms in the basic cell. The strain rate is specified as $\dot{\varepsilon }$ = 0.000833/τ in the *z* direction, which is the same as the simulation work from Aoyagi et al. [3]. The average stress σ in the *z* direction is obtained from the deviatoric part of the stress tensor σ = (1 + μ)(−*P_ZZ_* + *P*) ≈ *3*(−*P_ZZ_* + *P*)/2, where *P = Σ_i_P_ii_*/3 is the hydrostatic pressure [29,30,31]. The parameter μ stands for Poisson’s ratio, which is equal to 0.5 in our simulation. In order to quantitatively compare the viscoelasticity, we adopt the dynamic hysteresis loss (DHL), which is defined to be the ratio of the dissipated energy to the stored energy during the tension-recovery process in one cycle. For the oscillatory shear deformation, we use the so-called (SLLOD) equations of motion [32]. To realize the deformation process of the simulation box, we carry out the incremental deformation every time step, indicating that the deformation extent is proportional to the deformation time, which is implemented in the large-scale atomic/molecular massively parallel simulator (LAMMPS) package [33]. The upper *xy* plane of the simulation box is shifted along the *x* direction so that each point in the simulation box can be considered as having a “streaming” velocity. This position-dependent streaming velocity is subtracted from each atom’s actual velocity to yield a thermal velocity, which is used for temperature computation and thermostatting. The shear strain is defined as γ = δ*_x/_L_Z_(0)*, where the offset δ*_x_* is the transverse displacement distance in the shear direction (*x* direction for *xy* deformation) from the unstrained orientation, and *Lz(0)* is the box length perpendicular to the shear direction. Additionally, in most cases, the shear strain rate is around $\dot{\gamma }=0.01/\tau$. In our simulations, the maximum value of the shear strain amplitude was set to γ° = 1.0. The period of the oscillatory shear was varied from 25–200 τ, and thus, the corresponding frequency *ν* ranged from 0.04–0.005 in units of τ^−1^. The average shear stress is obtained from the deviatoric part of the stress tensor δ*_s_ = P_xy_ = P_yx_*. We further obtain the hysteresis loss by integrating the area of the hysteresis loop in one cycle.

All MD runs are carried out through the large-scale atomic/molecular massively parallel simulator (LAMMPS), which is developed by Sandia National Laboratories [34]. More simulation details can be found in our previous work [35,36,37].

## 3. Results and Discussion

### 3.1. Effect of the Structural Evolution of the ABA Tri-Block Copolymer

**Figure 1:**
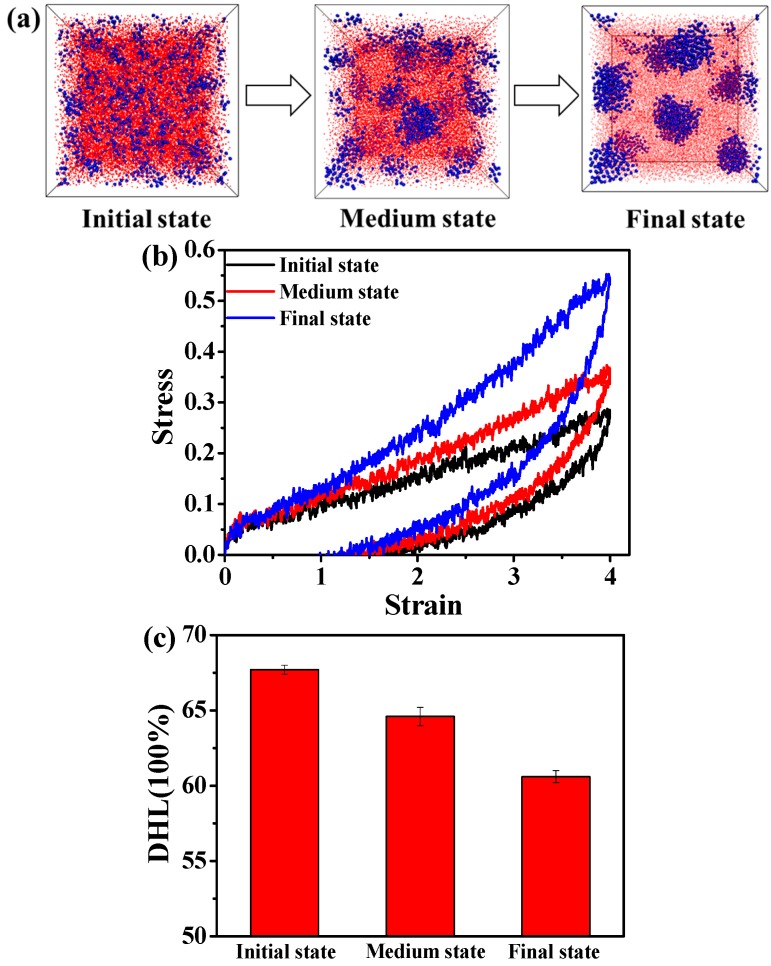
(**a**) The self-assembly process of the microstructure of the A_5_B_90_A_5_ tri-block copolymer. Note that the blue spheres represent the hard component of polymer chains, and the small red beads stand for the soft component of polymer chains. (**b**) The stress-strain behavior of the tri-block copolymer at three typical states; (**c**) the dynamic hysteresis loss (DHL) derived from the tension-recovery process.

We firstly examine the effect of the volume fraction of A blocks (f_A_) on the morphology of the ABA tri-block copolymer. Based on the previous literature, the A_5_B_90_A_5_ tri-block copolymer with f_A_ = 0.1 tends to self-assemble to gradually form the spherical domains, as shown in Figure 1a. We study the stress-strain behavior during the self-assembly process. We mainly consider three typical cases, termed as the initial state, medium state and final state, respectively, in the process of self-assembly. From Figure 1b, we can observe that the stress-strain behavior is enhanced as the ordered structure is gradually developed. It is known that the TPEs are a kind of material with a self-reinforcing effect. The glassy domains formed by A blocks act as the reinforcing regions. As shown in Figure 1b, the more ordered the glassy domains, the greater the stress-strain curve. Furthermore, to examine the effect of hard domains formed by A blocks on the DHL, we focus our attention on the permanent set of the tension-recovery process [29]. Theoretically, a large permanent set always means great slippage and internal friction between elastomeric macromolecule chains, consequently resulting in more hysteresis loss [38]. We observe that the permanent set gradually decreases from the initial state to the final state, as presented in Figure 1b. Moreover, we also calculate the DHL, as displayed in Figure 1c. Obviously, the TPEs with the ordered state exhibit the smallest DHL.

**Figure 2:**
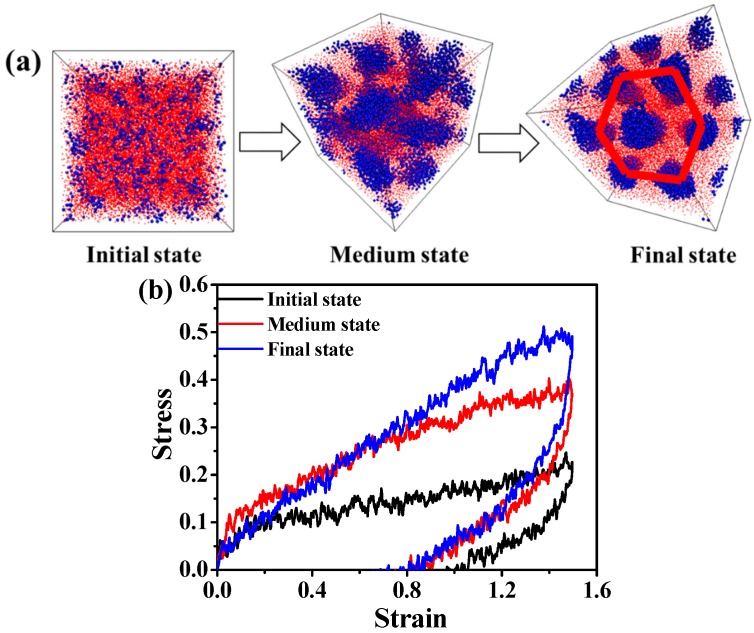

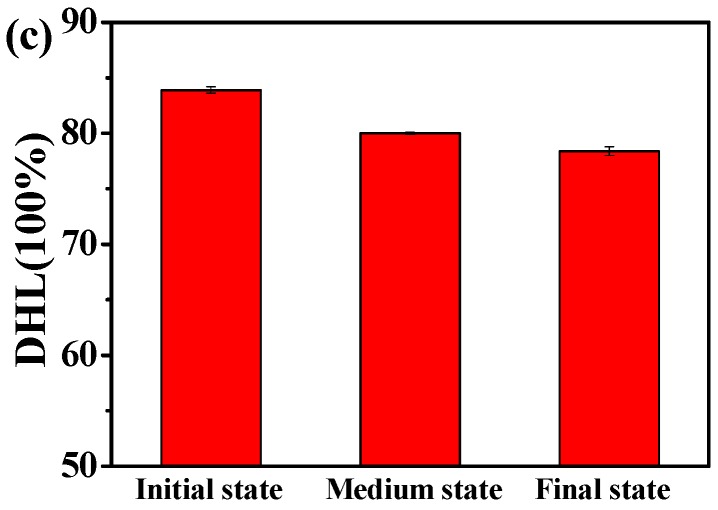
(**a**) The self-assembly process of the microstructure of the A_5_B_20_A_5_ tri-block copolymer. Note that the blue spheres represent the hard component of polymer chains, and the small red beads stand for the soft component of polymer chains. The red circle means forming a hexagonally cylindrical ordered structure; (**b**) The stress-strain behavior of the tri-block copolymer at three typical states; (**c**) The dynamic hysteresis loss (DHL) derived from the tension-recovery process.

**Figure 3:**
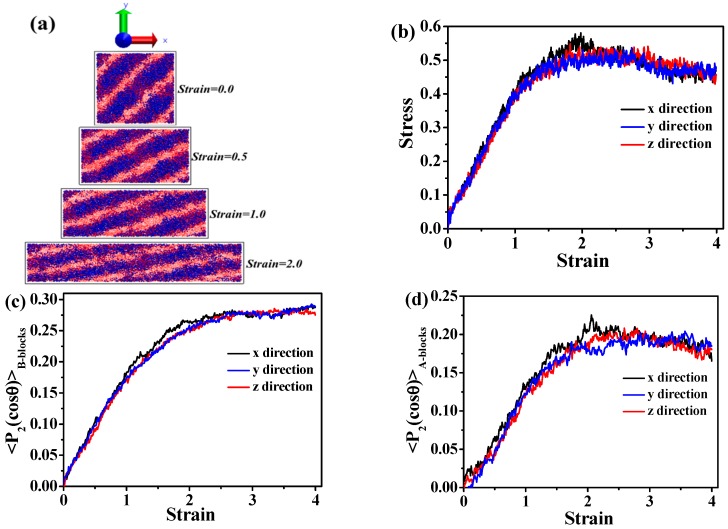
(**a**) The microstructure evolution of the A_5_B_20_A_5_ tri-block copolymer during the tension process in the *x* direction; (**b**) the stress-strain behavior of the tri-block copolymer at three different directions. The bond orientation of (**c**) B blocks and (**d**) A blocks during the tension process.

**Figure 4:**
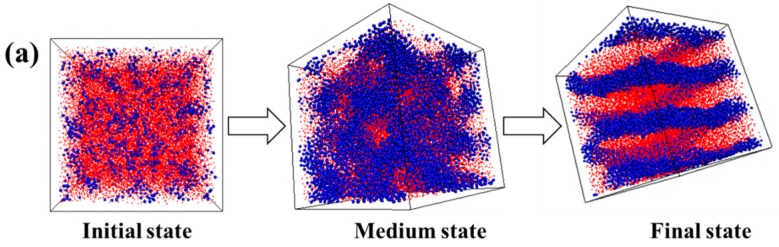

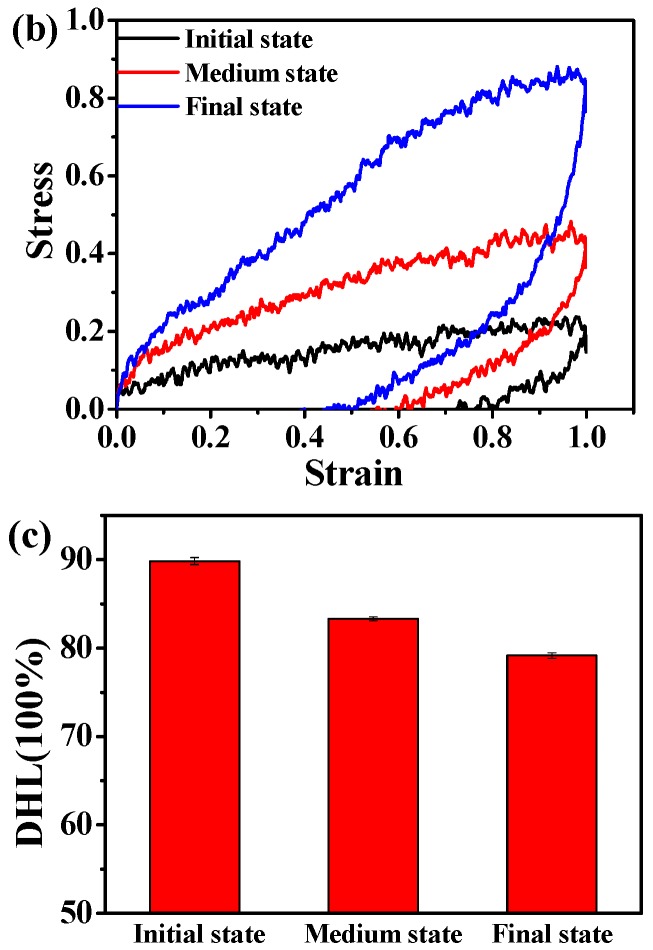
(**a**) The self-assembly process of the microstructure of the A_5_B_10_A_5_ tri-block copolymer. Note that the blue spheres represent the hard component of polymer chains, and the small red beads stand for the soft component of polymer chains. (**b**) The stress-strain behavior of the tri-block copolymer at three typical states. (**c**) The dynamic hysteresis loss (DHL) derived from the tension-recovery process.

**Figure 5:**
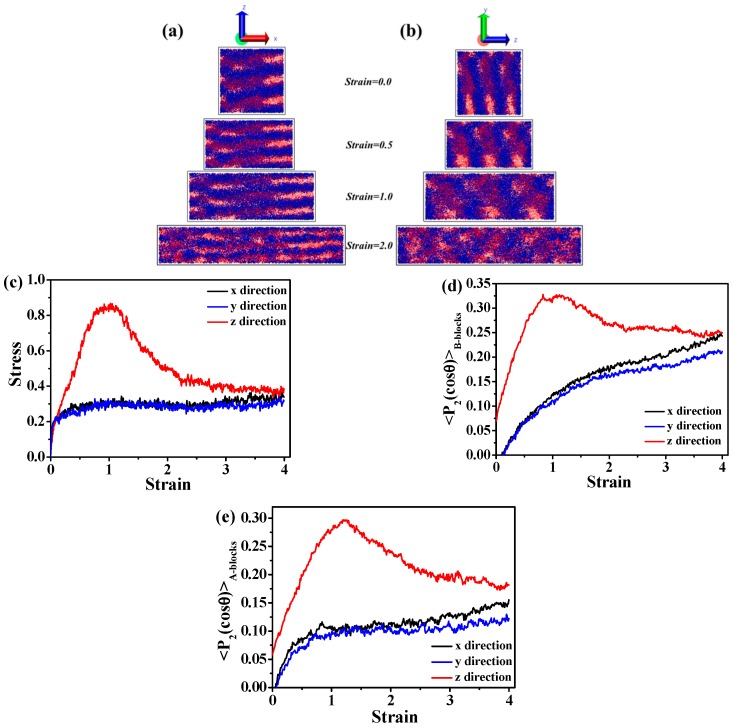
The microstructure evolutions of the A_5_B_10_A_5_ tri-block copolymer during the tension process in the (**a**) *x* direction and (**b**) *z* direction; (**c**) the stress-strain behavior of the tri-block copolymer at three different directions; the bond orientation of (**d**) B blocks and (**e**) A blocks during the tension process.

Later on, we increase the volume fraction of A blocks. We observe that the A_5_B_20_A_5_ tri-block copolymer with f_A_ = 0.33 gradually forms a hexagonally cylindrical ordered structure, as shown in Figure 2a. We also consider the stress-strain behavior, as well as the DHL from the initial state to the final state, as displayed in Figure 2b,c. We obtain nearly the same result as in the case of the A_5_B_90_A_5_ tri-block copolymer. In theory, the mechanical property of the system with spherical domains should be isotropic, while that of other systems could be anisotropic, because of the orientated self-assembled structures, such as cylindrical and lamellar domains. The microstructural evolution of the cylindrical domain-filled system during the tensile process along the *x* direction is displayed in Figure 3a. It is observed that the cylindrical domains are orientated along the tensile direction. Since the cylindrical domains are initially dispersed randomly, this leads to the fact that the stress-strain behavior exhibits isotropic behavior, as shown in Figure 3b. The bond orientation of B blocks and A blocks along the tensile direction further verifies the stress-strain behavior, as illustrated in Figure 3c,d. Lastly, we study the self-assembly behavior of the A_5_B_10_A_5_ tri-block copolymer with f_A_ = 0.5, as shown in Figure 4a, indicating that A blocks gradually self-assemble to from a lamellar structure. We further study the stress-strain behavior and the DHL, as presented in Figure 4b,c. Similar to the self-assembled structures, like spherical and cylinder domains, the more ordered the lamellar structure, the more significant the stress-strain behavior becomes in Figure 4b. Meanwhile, the well-ordered state also exhibits the smallest DHL, in accordance with the previous two systems. Moreover, in Figure 4a, we observe that the lamellar domains are parallel to the *x* and *y* directions and perpendicular to the *z* direction. We test the stress-strain behavior at all three directions; the snapshots are displayed in Figure 5a,b. It is observed that the evolution of lamellar domains is totally different along the *x* and *z* directions, leading to different stress-strain curves, as shown in Figure 5c. A bigger system (48,000 beads) shown in Figure S3 was further explored to verify the above-mentioned stress-strain behavior, as displayed in Figure S4. We infer that there may exist buckling instability perpendicular to the lamellae domains, which is consistent with the discoveries from Honeker et al. [39]. In their literature, they expediently discover that if polystyrene-polyisoprene-polystyrene (SIS) or poly(styrene-butadiene-styrene) (SBS) copolymers in a globally-aligned hexagonal or lamellar phase are stretched perpendicular to the rods or plates, then above a critical strain, they buckle to form a “chevron” morphology. The morphology can be seen in electron micrographs and is also characterized by “four point” patterns in the X-ray scattering. The bond orientation of B blocks and A blocks along the tensile direction further supports the stress-strain behavior in Figure 5d,e. In general, when the ordered structure is changed from the spherical domain to the lamellar domain, the stress-strain behavior is gradually enhanced. The stress-strain curve is enhanced, and the DHL is reduced with the gradual formation of the order domains.

**Figure 6:**
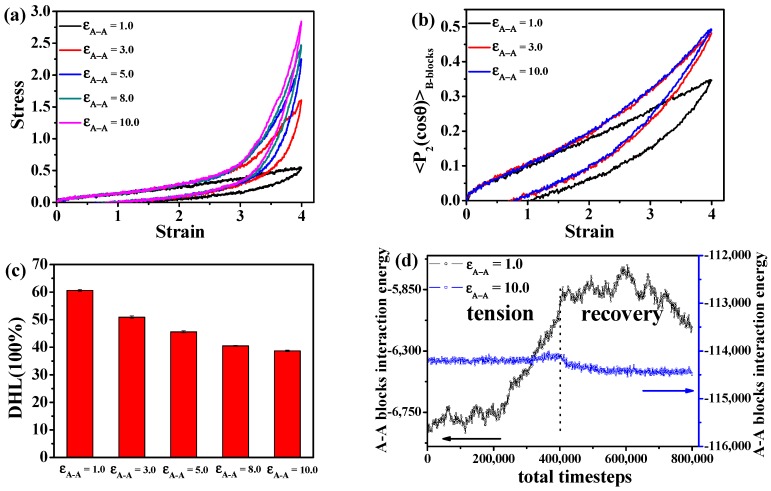
The effect of the interaction strength between A-A blocks (ε_A-A_) (**a**) on the tension-recovery stress-strain behavior and (**b**) the bond orientation of B blocks during the tension-recovery process; (**c**) dynamic hysteresis loss (DHL) derived from the tension-recovery process for various interaction strength between A-A blocks; (**d**) the change of the total A-A blocks interaction energy during the tension-recovery process.

As we all know, TPEs are a kind of elastomeric material composed of hard and soft segments. It is evident that the interaction strength between the hard segments affects the static and dynamic mechanical properties. In theory, the more stable the structure of the uniformly-dispersed nano-domains, the smaller the DHL, and this stability is directly related to the interaction strength between A-A blocks. To verify this assumption, we mainly consider the case of spherical domains, we change the interaction strength between A-blocks (ε_A-A_) ranging from 1.0–10.0, and after enough equilibration, we perform the tension-recovery test, as shown in Figure 6a. Obviously, with the increase of ε_A-A_, the stress-strain behaviors are enhanced, exhibiting better mechanical reinforcement. For instance, in comparison with the system of ε_A-A_ = 1.0, the stress at the strain ε = 4.0 for the system with ε_A-A_ = 10.0 is almost five-times greater. Meanwhile, it is observed that the permanent set is also gradually decreasing, which could be attributed to the fact that the stable domains formed by A-blocks lead to better orientation-disorientation of the B blocks during the tension-recovery process. In order to verify this assumption, here, we use the second-order Legendre polynomials <*P*_2_(cosθ)> to characterize the bond orientation as follows.

(3) $$\langle P_{2}\left(\cos\theta \right)\rangle =\left(3\langle \cos^{2}\theta \rangle -1\right)/2$$

where θ denotes the angle between a given element (two adjoining monomers in the chain) and the reference direction, which is referred to the stretching direction. The possible values of <*P*_2_(cosθ)> range from −0.5–1, and <*P*_2_(cosθ)> = −0.5, 1.0, 0.0 each indicates a perfect orientation perpendicular to the reference direction, parallel to the reference direction or randomly oriented. Obviously, from Figure 6b, the polymer chains of B-blocks exhibit higher orientation-disorientation with the increase of ε_A-A_. In order to further examine the quantitative effect of the interaction energy between A blocks ε_A-A_ on the visco-elasticity, we can directly calculate the DHL from the tension-recovery cycle, as shown in Figure 6c. The DHL gradually decreases with the increase of ε_A-A_. In all, the higher ε_A-A_, the better static and dynamical mechanical properties of the TPEs.

**Figure 7:**
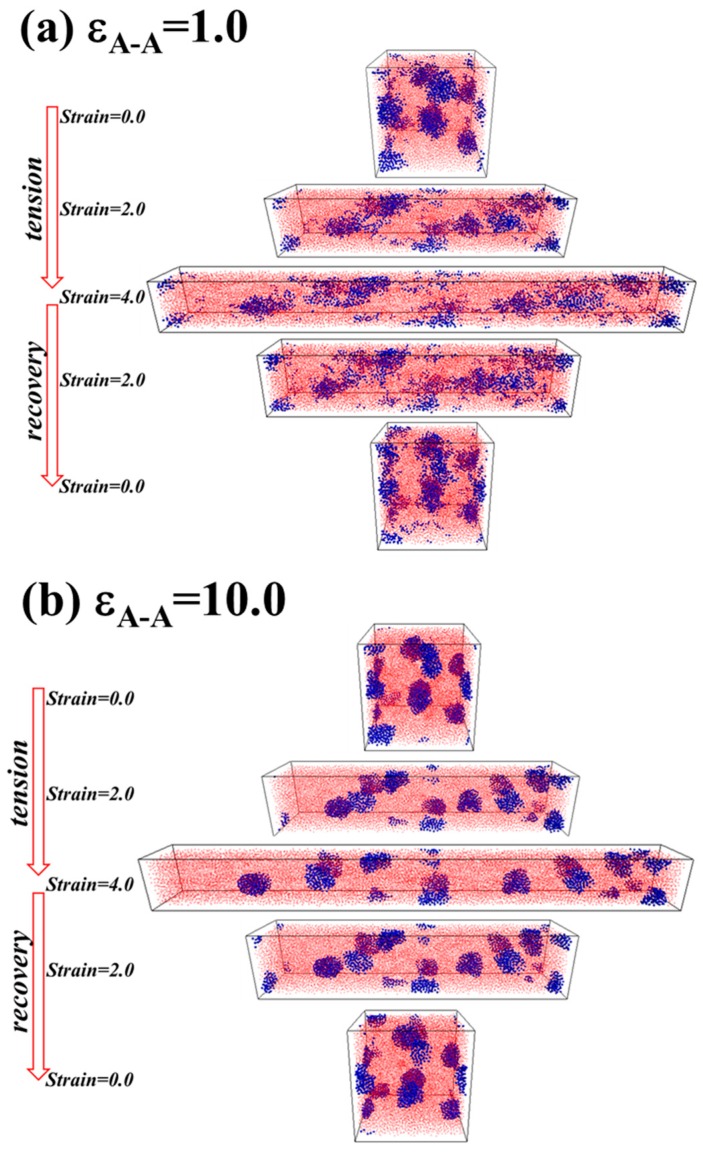
The microstructure evolution of the A_5_B_90_A_5_ tri-block copolymer during the tension-recovery process at: (**a**) ε_A-A_ = 1.0; (**b**) ε_A-A_ = 10.0. Note that the blue spheres represent the hard component of polymer chains, and the small red beads stand for the soft component of polymer chains.

In order to further explain the effect of ε_A-A_ on the static and dynamical mechanical properties, we analyze the microstructure evolution during the tension-recovery process. Firstly, we directly calculate the total interaction energy between A-A blocks, as presented in Figure 6d. Clearly, in the case of ε_A-A_ = 1.0, the absolute value of the total interaction energy between A-A blocks decreases during the tensile deformation, which means the hard domains formed by A blocks gradually become broken up, although they recover slightly during the recovery process. This observation directly confirms that the damage behavior of the hard domains is irreversible. However, the absolute value of the total interaction energy between A-A blocks remains essentially constant, meaning that the hard domains keep stable during the tension-recovery process in the case of ε_A-A_ = 10.0. In addition, we examine the microstructure evolution of the TPEs during the tension-recovery test, and the deformed snapshots are displayed in the case of ε_A-A_ = 1.0 and ε_A-A_ = 10.0 in Figure 7a,b. Evidently, the hard domains formed by A blocks become gradually broken and slightly recover in the case of ε_A-A_ = 1.0, while the hard domains keep stable in the case of ε_A-A_ = 10.0 during the tension-recovery process. It is concluded that the much stronger interaction strength between A-A blocks leads to a more stable network structure during the deformation, leading to better stress-strain performance and less DHL.

### 3.2. Effect of the Temperature

**Figure 8:**
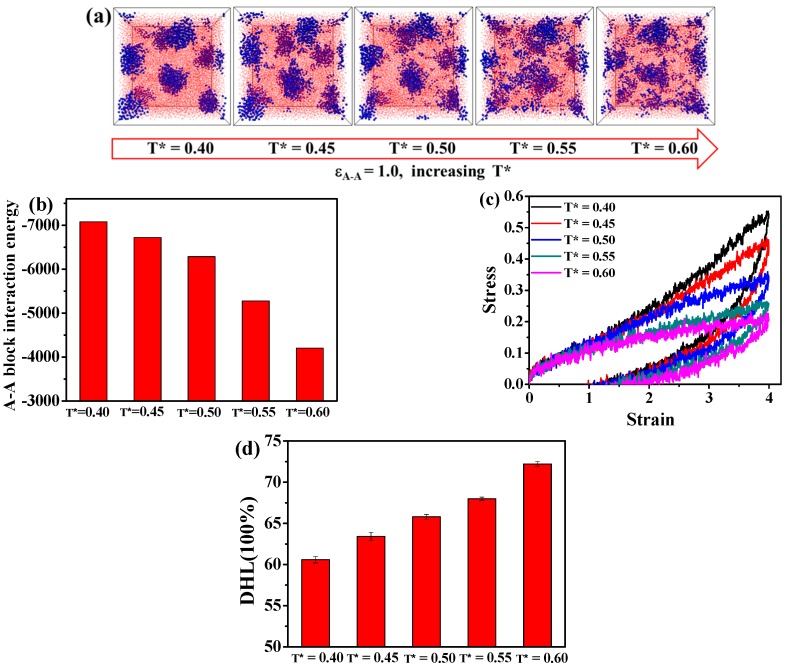
(**a**) The effect of the temperature on the microstructure evolution of the A_5_B_90_A_5_ tri-block copolymer ranging from T* = 0.40 to T* = 0.60 in the case of ε_A-A_ = 1.0. Note that the blue spheres represent the hard component of polymer chains, and the small red beads stand for the soft component of polymer chains. (**b**) The change of the total A-A blocks interaction energy at different equilibrated temperature; the effect of the temperature on (**c**) the tension-recovery stress-strain behavior and (**d**) the dynamic hysteresis loss (DHL).

In this part, we turn to examine the effect of the temperature on the morphology of the A_5_B_90_A_5_ tri-block and the resulting mechanical properties. As we know, TPEs are a kind of elastomeric material, whose properties depend on temperature [40]. In theory, as the temperature increases, the domains made up of hard segments tend to get broken, therefore affecting the mechanical properties, which, however, is difficult to study in experiments. In order to verify this consumption, we analyze the microstructure evolution with the increase of the temperature, as illustrated in Figure 8a. We get that the hard domains are gradually destroyed as the temperature increases ranging from T* = 0.40 to T* = 0.60 in the case of ε_A-A_ = 1.0. We further calculate the total interaction energy between A-A blocks to quantitatively analyze this microstructure evolution, as shown in Figure 8b. It is evident that the increase of the temperature leads to the damage of domains formed by A blocks. This phenomenon is consistent with the experimental work from Neumann et al. [41]. They get that the corresponding poly(ethylene-alt-propylene)-block-poly(ethylene-co-butylene) di-block copolymers show an order-disorder transition in the range of 10–20 °C.

Following this, we examine the mechanical properties of the TPEs equilibrated at different temperatures, as presented in Figure 8c. We can find that the stress-strain behavior and the permanent set decrease moderately with the increase of the temperature; since the stable domains formed by A blocks are damaged severely as the temperature increases, leading to less reinforcing effect and higher polymer friction during the tension-recovery process. Lastly, we calculate the DHL in the case of different temperature, as displayed in Figure 8d. Generally, the DHL gradually increases with the damage of the hard domains.

**Figure 9:**
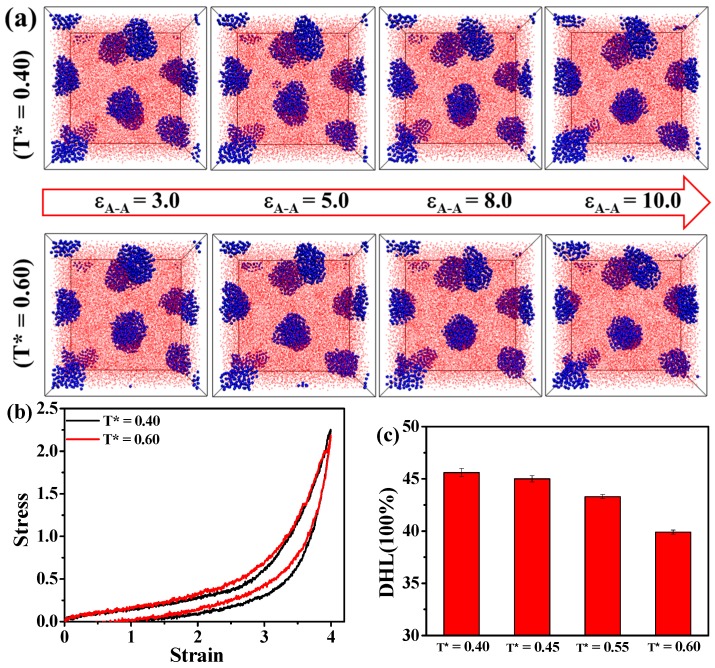
(**a**) The effect of the temperature on the microstructure evolution of the A_5_B_90_A_5_ tri-block copolymer ranging from T* = 0.40 to T* = 0.60 in the case of ε_A-A_ equal or greater than 3.0; the effect of the temperature on (**b**) the tension-recovery stress-strain behavior and (**c**) the dynamic hysteresis loss (DHL) in the case of ε_A-A_ = 5.0. Note that the blue spheres represent the hard component of polymer chains, and the small red beads stand for the soft component of polymer chains.

We also study the effect of the temperature on the TPEs at different A-A blocks’ interaction energy ε_A-A_, as presented in Figure 9a. Obviously, when ε_A-A_ is above or equal to 3.0, the hard domains can be maintained stable in a specific temperature range from 0.40–0.60. We further calculate the stress-strain behavior at different temperatures in the case of ε_A-A_ = 5.0 in Figure 9b. We find that the TPEs can maintain the mechanical performance at a stronger interaction strength between A-A blocks. Furthermore, we calculate the DHL of TPEs at ε_A-A_ = 5.0, as shown in Figure 9c. Interestingly, the DHL decreases as the temperature increases. We explain this observation as follows: the elasticity of TPEs totally results from the entropy of polymer chains, and the entropy of polymer chains increases with the increase of the temperature. As we know, the TPEs can maintain the perfect network structure in the case of ε_A-A_ above or equal to 3.0. Therefore, the TPEs can exhibit good mechanical properties and less DHL.

In all, the results indicate that TPE is a strong temperature-dependent material in the case of weak interaction strength between A-A blocks, such as ε_A-A_ = 1.0. As the temperature increases, the microstructure of the domains is gradually damaged, corresponding to the deterioration of the mechanical properties and higher DHL. However, in the case of ε_A-A_ above or equal to 3.0, the TPEs become more temperature-resistance. Additionally, the TPEs can maintain good mechanical properties and behave with less DHL as the temperature increases from T* = 0.40 to T* = 0.60.

### 3.3. Effect of the Dynamic Shear Flow

**Figure 10:**
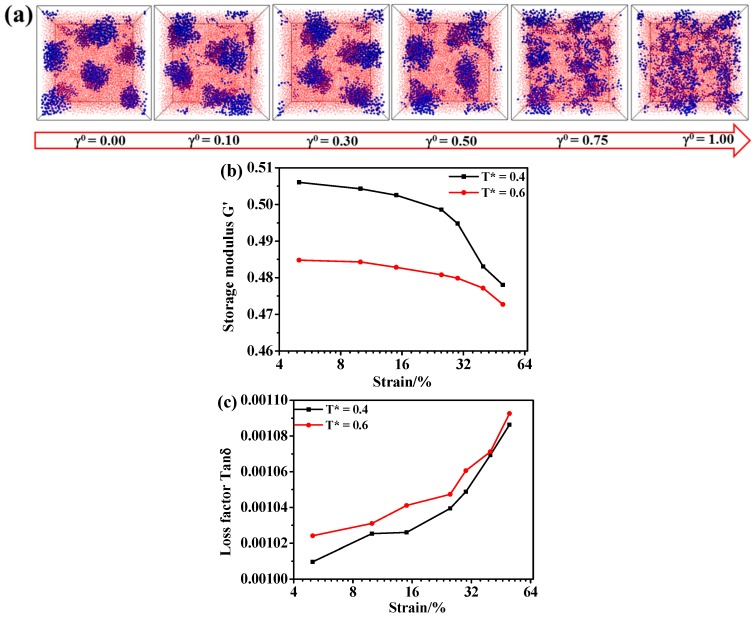
(**a**) The effect of the shear amplitude on the microstructure evolution of the A_5_B_90_A_5_ tri-block copolymer in the case of the shear frequency equal to 0.01. Note that the blue spheres represent the hard component of polymer chains, and the small red beads stand for the soft component of polymer chains; (**b**) The storage modulus G′ as a function of the shear strain; (**c**) The loss factor tan δ as a function of the shear strain.

Lastly, we extend our efforts to study the effect of the dynamic shear on the morphology of the A_5_B_90_A_5_ tri-block. As we know, except for the effect of the temperature, the dynamic shear is another factor affecting the microstructure evolution of the hard domains [42,43,44]. For the oscillatory shear, two independent factors are involved: the strain amplitude and the shear frequency. Hence, we firstly fix the shear frequency at ν = 0.01 to examine the effect of the strain amplitude in the range of 0.05–1.0 for the case of T* = 0.40, as displayed in Figure 10a. The snapshots show that the microstructure of the hard domains remains relatively stable under the amplitude lower than γ° = 0.30. When the shear amplitude becomes equal to or greater than 0.30, the hard domains are gradually broken up.

**Figure 11:**
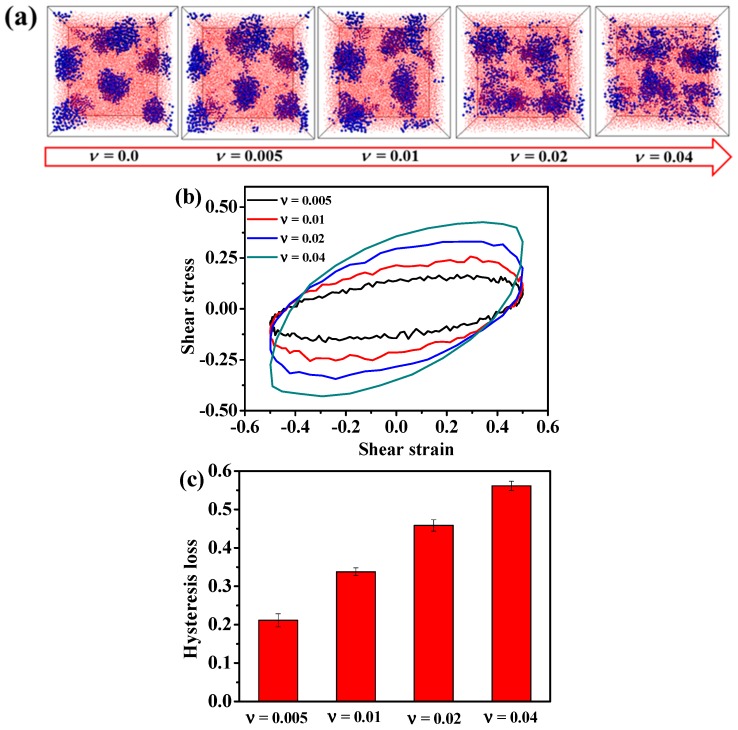
(**a**) The effect of the shear frequency on the microstructure evolution of the A_5_B_90_A_5_ tri-block copolymer in the case of shear amplitude equal to 0.50. Note that the blue spheres represent the hard component of polymer chains, and the small red beads stand for the soft component of polymer chains. (**b**) The dynamic hysteresis loop at different shear frequencies. (**c**) The hysteresis loss integrated from (**b**) for various shear frequencies.

We further calculate the storage modulus G′ as a function of the shear strain, as illustrated in Figure 10b. Interestingly, according to Figure 10b, starting at a small shear strain of 0.05, the storage modulus of this TPE in the case of ε_A-A_ = 1.0 first exhibits a plateau at the shear amplitude lower than 0.30, then decreases with the increase of the shear amplitude for both T* = 0.40 and T* = 0.60. Since the hard domains are already broken to a certain degree at the temperature of T* = 0.60 (as shown in Figure 8a), the storage modulus G′ of the system T* = 0.60 is a little lower than that of T* = 0.40. Moreover, we calculate the loss factor tan δ as a function of the shear amplitude, as displayed in Figure 10c. Obviously, there also exists a plateau at the low shear amplitude, and it increases dramatically when the shear amplitude is greater than 0.30. Attributed to the damage of the hard domains at T* = 0.60, the loss factor tan δ of the system T* = 0.60 is higher than that of T* = 0.40 because of the strong polymer chain frictions. In all, the storage modulus G′ and the loss factor tan δ are consistent with the microstructural evolution of the hard domains during the dynamic shear process discussed above. Namely, the more stable structure of the hard domains could lead to higher storage modulus G′ and lower loss factor δ. Qualitatively, this non-linear characteristic is very similar to the experimentally-observed “Payne effect”. Particularly, the change of the storage modulus in the presence of the hard domains is more sensitive to the imposed shear strain, by comparing the case of T* = 0.40 to T* = 0.60. Next, we examine the effect of the shear frequency on the morphology of the TPEs. Figure 11a presents the resulting structures of the TPEs under the condition that the shear frequency is varied from 0.005 to 0.04 with the strain amplitude being fixed at γ° = 0.50 in the case of T* = 0.40. Obviously, the shear frequency shows the same results as those of the strain amplitude, that is the structure first becomes stable at lower shear frequency and then evolves into a disordered one. This result is line with the discovery of Cui et al. [45]. In their study, both simulative and experimental results show that a higher shear rate results in the bigger domains becoming the smaller vesicles. Here, we further consider the effect of the shear frequency on the hysteresis loop, as displayed in Figure 11b. In order to further quantitatively compare the effect of the shear frequency on the visco-elasticity, we can directly calculate the hysteresis loss, namely the area of the hysteresis loop in one cycle. Figure 11c shows that the hysteresis loss gradually increases with the increase of shear frequency, which can be attributed to the fact of the severely damaged hard domains in the case of the higher shear frequency. Clearly, the hard domains of the TPEs can remain stable in an optimal combination of the strain amplitude and frequency, leading to the TPEs exhibiting higher storage module G′ and lower loss factor tan δ. Namely, the higher shear amplitude and shear rate lead to the phase evolution, which is considered to be the primary source of the hysteresis loss. In general, this finding is consistent with the experimental observation work of Qi et al. [14].

## 4. Conclusions

In this work, we investigate the morphology, the static and dynamic mechanical properties of the TPEs through molecular dynamics simulation. With the increase of the volume fraction of A blocks, the TPEs exhibit three typical states: spherical, cylindrical and lamellar phases. During the self-assembly process, the stress-strain behavior is gradually enhanced with the formation of the ordered structures. The spherical or cylindrical domain-filled systems exhibit isotropic mechanical properties, while the lamellar domains filled system displays totally different stress-strain behavior along the direction parallel and perpendicular to the lamellar phases. The strong interaction strength between A-A blocks enhances the stress-strain behavior and results in less dynamic hysteresis loss. We further consider the effect of the temperature on the morphology and mechanical properties. The results indicate that the TPEs are strongly temperature-dependent in the case of ε_A-A_ = 1.0, and this exhibits more temperature resistance when ε_A-A_ becomes equal or greater than 3.0. Lastly, the effect of the dynamic shear on the TPEs is also investigated. It is verified that the hard domains of the TPEs can remain stable in an optimal combination of the strain amplitude and frequency, leading to the TPEs exhibiting higher storage modulus G′ and the lower loss factor tan δ. Qualitatively, these non-linear characteristics are very similar to the experimentally-observed “Payne effect”.

## Figures and Tables

**Table 1:** The Lennard–Jones interaction strength between A-A, B-B and A-B blocks.

Pair of Beads	ε	σ	*r_cutoff_*
A-A	1.0	1.0	2.5
A-B	1.0	1.0	1.5
B-B	1.0	1.0	2^1/6^

## Supplementary Materials

The following are available online at www.mdpi.com/2073-4360/8/9/335/s1, Figure S1: Plot of the specific volume as a function of the temperature for the pure system of A blocks, Figure S2: After enough equilibration (1 × 10^8^ MD steps) with the NVT ensemble, the change of the potential energy, the variation of the mean squared end-to-end distance $R_{\mathrm{end}}^{2}$, and the radius of gyration $R_{g}^{2}$ of all three systems in the following 1 × 10^6^ MD steps, Figure S3: The snapshot of the A_5_B_10_A_5_ tri-block copolymer with 48,000 beads, Figure S4: The stress-strain behavior of the A_5_B_10_A_5_ tri-block copolymer (48,000 beads) in three different directions.
