# Supplementary material for: Tailoring the Static and Dynamic Mechanical Properties of Tri-Block Copolymers through Molecular Dynamics Simulation
Source: Polymers (Basel). 2016 Sep 19;8(9):335. doi: 10.3390/polym8090335 (PMC6432360; doi:10.3390/polym8090335)
Supplement: Supplementary file 1 [file polymers-08-00335-s001.pdf]

Supplementary Materials: Tailoring the Static and Dynamic Mechanical Properties of Tri-Block Copolymers through Molecular Dynamics Simulation

Zijian Zheng, Hongji Liu, Jianxiang Shen, Jun Liu, Youping Wu and Liqun Zhang

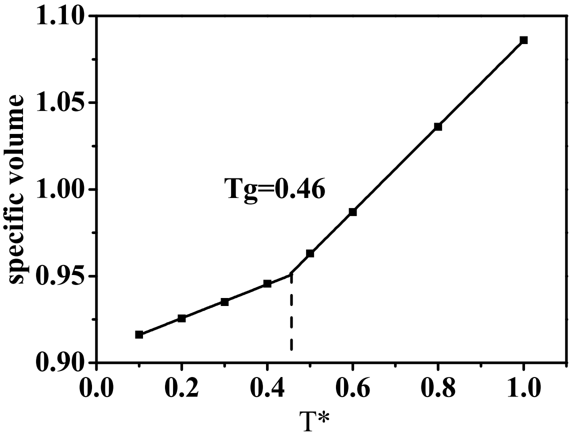

Figure S1. Plot of the specific volume as a function of the temperature for the pure system of A blocks.

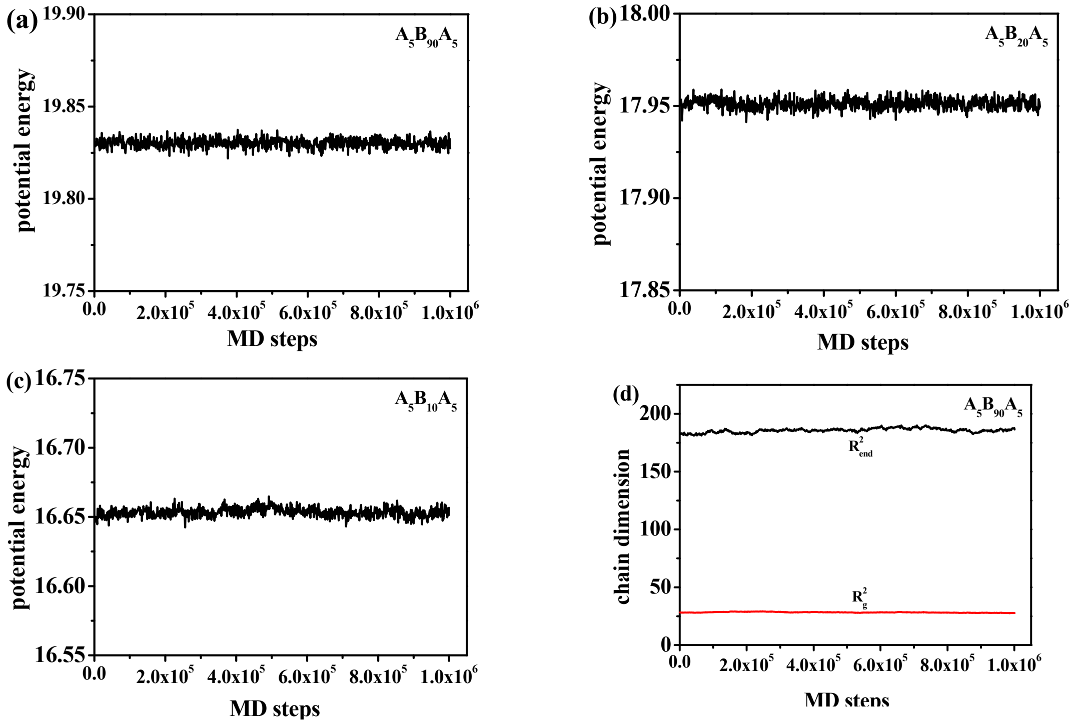

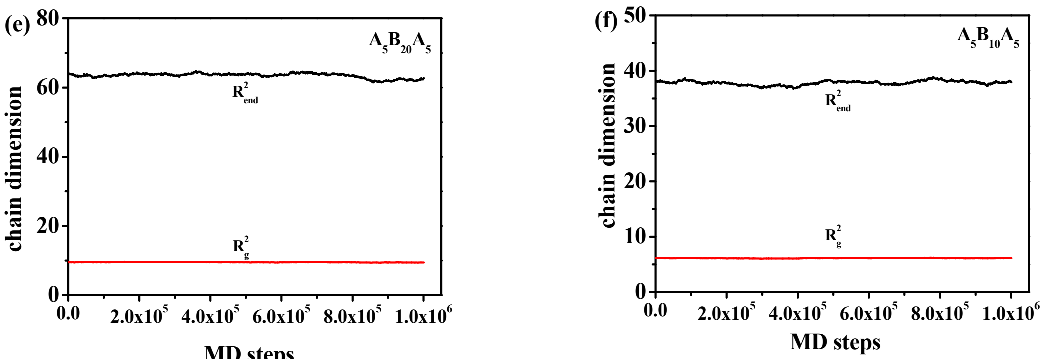

Figure S2. After enough equilibration (1 × 10^8 MD steps) with the NVT ensemble, the change of the potential energy (**a**–**c**), the variation of the mean squared end-to-end distance R^2_{end}, and the radius of gyration R^2_g of all three systems in the following 1 × 10^6 MD steps (**d**–**f**).

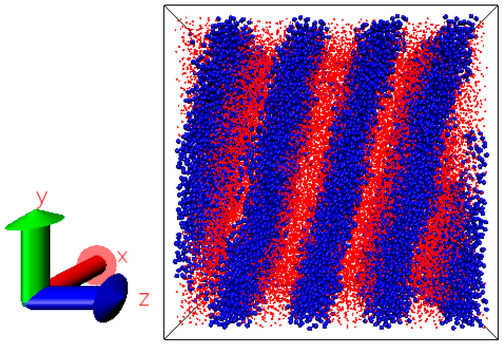

Figure S3. The snapshot of the $A_5B_{10}A_5$ tri-block copolymer with 48,000 beads.

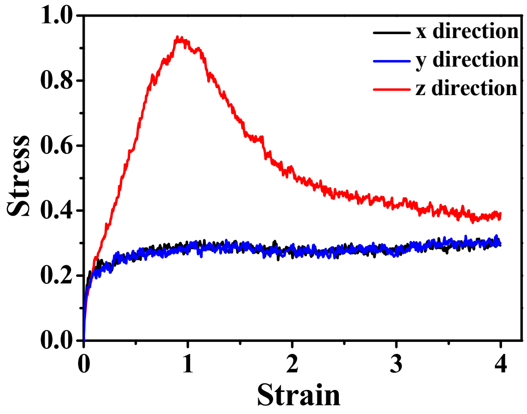

Figure S4. The stress-strain behavior of the $A_5B_{10}A_5$ tri-block copolymer (48,000 beads) in three different directions.

1. Further Discussion

We have performed the case with a much longer chain length, which varies from $A_{16}B_{67}A_{17}$ to $A_{25}B_{50}A_{25}$, corresponding to the formation of the cylindrical and lamellar phases, respectively. The results are shown in Figures S5a and S6a. Obviously, the stress-strain behavior of these two systems exhibits anisotropic behavior, as displayed in Figures S5b and S6b.

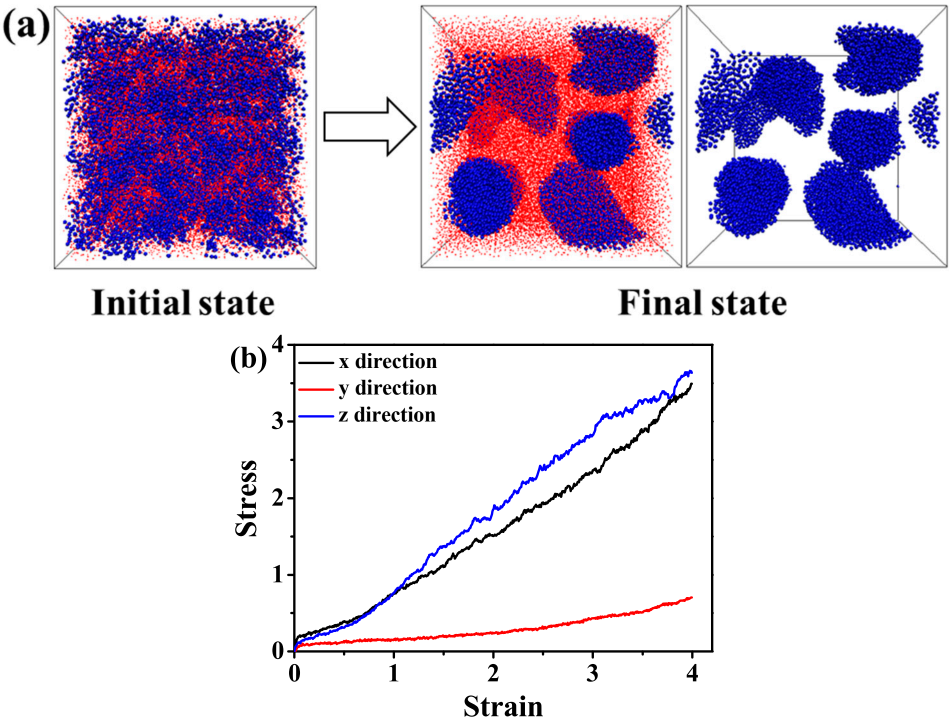

Figure S5. (**a**) The self-assembly process of the microstructure of A16B63A17 tri-block copolymer; (**b**) The stress-strain behavior of the tri-block copolymer along three different directions.

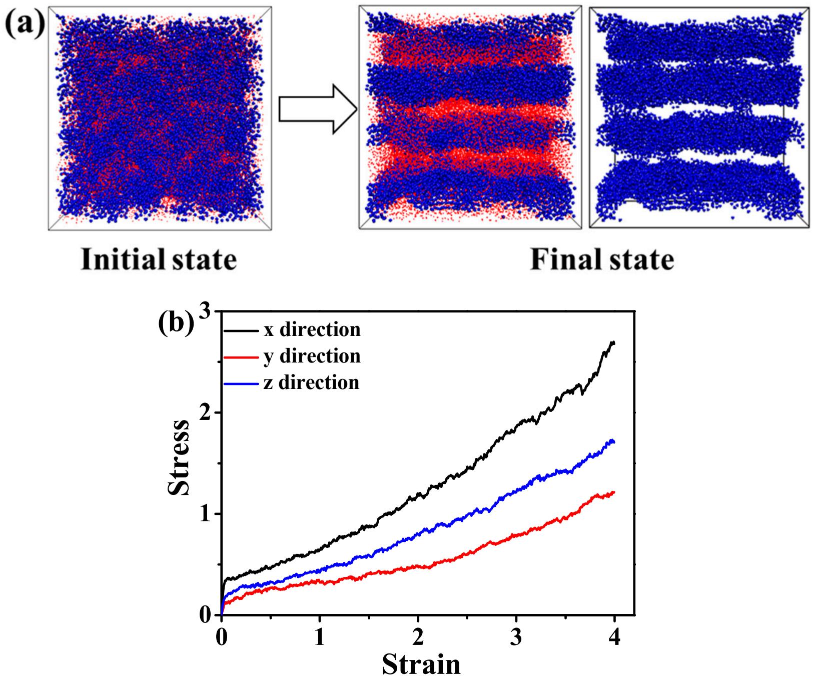

Figure S6. (**a**) The self-assembly process of the microstructure of A25B50A25 tri-block copolymer; (**b**) The stress-strain behavior of the tri-block copolymer along three different directions.
